# Graphene-Based Materials, Their Composites, and Potential Applications

**DOI:** 10.3390/ma15207184

**Published:** 2022-10-15

**Authors:** Maria Cristina Ramirez, Maria Isabel Osendi

**Affiliations:** Institute of Ceramics and Glass, Spanish National Research Council (CSIC), 28049 Madrid, Spain

Since its isolation in 2004, monolayer graphene has attracted enormous attention within the scientific community, the industry, and the general public owing to its exceptional properties (electrical, optical, thermal, and mechanical) and prospects [1,2]. A full understanding of its fundamental physics and properties has been gained, as well as a significant advancement in scaling up the production methods [3,4]. In parallel, new routes for the preparation of bulk porous graphene materials and foams that envisage fascinating applications in areas such as environmental science, bio-medicine, and energy have steadily grown [5,6].

**Figure 1:**
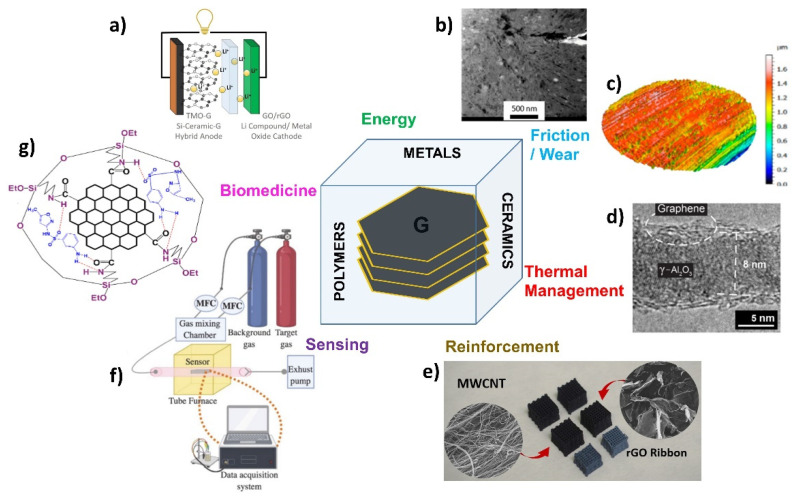
A schematic diagram of the graphene composites spectrum and their potential uses along with representative examples of materials and uses from the articles included in this Special Issue. (**a**) A schematic of the applications of graphene-based composites in Li-ion battery electrodes described in [11]; (**b**) a high annular dark field (HAADF) micrograph of an Al/2GNP/1Al_2_O_3_ composite [13]; (**c**) an isometric view of a PAO8/ZDDP/graphene W-DLC surface after a ball-on-disc test [14]; (**d**) CVD graphene-coated γ-Al_2_O_3_ nanofiber [15]; (**e**) reinforced 3D-printed MWCNT and rGO/Al_2_O_3_ scaffolds [16]; (**f**) a schematic of an experimental setup for gas-sensing applications [10]; (**g**) a schematic diagram of sulfamethoxazole detection by silica-coated GQDs [12].

Graphene-based composites, often containing multilayer types of graphene of different sizes and several defects, is also a bustling field of research due to the boosting properties that this filler offers to matrix materials such as polymer, metal, or ceramics [7,8,9]. This Special Issue presents a view of the current research on graphene-based composites with applications in diverse fields (Figure 1), such as energy production and storage, environmental and friction protection, catalysis, biomedicine, and wearable electronic and sensing devices.

In particular, it offers two review papers, one from Zamri and Haseeb on the application of graphene/conductive polymer composites such as chemiresistive sensors focusing on the preparation methods and sensing performance of these composites [10], and the other by Ramírez et al. [11] on the variety of applications of comparatively lesser known graphene/ceramic composites, each giving full insides and prospectives for those composites. The article by Le et al. [12] develops a fluorescence sensor for the detection of antibiotics (sulfamethoxazole) based on graphene quantum dots (GQDs) entrapped in a molecularly imprinted silica polymer that can be applied in biomedical and environmental systems. 

Two articles deal with the effect of graphene nanoplatelets over the friction and wear properties of materials; both are key characteristics from the industrial point of view. The work of Omrani et al. [13] highlights the effect of multilayer graphene sheets as a solid lubricant for Al and Al/alumina composites, whereas the article by Kowalczyk et al. focuses on the interactions between graphene and a common lubricant, ZDDP (zinc dialkyldithiophosphate), for reducing friction and wear in both bare and DLC-coated steels [14]. 

The article by Saffar Shamshirgar [15] studies the thermoelectric effect of composites containing graphene-coated γ- Al_2_O_3_ fibers that were coated by CVD methods, evidencing the notable increase in the thermopower factor for specific compositions and, accordingly, their potential use for waste heat conversion.

Finally, the paper of Ramírez et al. [16] deals with the reinforced effect of graphene-type fillers on porous Al_2_O_3_ materials, in particular, γ- and α-Al_2_O_3_ lattices prepared by additive manufacturing methods, stressing the crucial reinforcing effect of GO nanoribbons over other fillers like CNT.
